# Social support needs for equity in health and social care: a thematic analysis of experiences of people with chronic fatigue syndrome/myalgic encephalomyelitis

**DOI:** 10.1186/1475-9276-10-46

**Published:** 2011-11-02

**Authors:** Jose C de Carvalho Leite, Maria de L Drachler, Anne Killett, Swati Kale, Luis Nacul, Maggie McArthur, Chia Swee Hong, Lucy O'Driscoll, Derek Pheby, Peter Campion, Eliana Lacerda, Fiona Poland

**Affiliations:** 1 School of Allied Health Professions, University of East Anglia, Norwich, England, United Kingdom, NR4 7TJ, UK; 2 London School of Hygiene and Tropical Medicine, Keppel Street, London, England, United Kingdom, WC1E 7HT, UK; 3 Faculty of Society and Health, Buckinghamshire New University, Uxbridge Campus, 106, Oxford Road, Uxbridge, Middlesex, UB8 1NA, England, UK; 4 Sports Therapy and Physiotherapy Division, University of Bedfordshire, Park Square, Luton, Bedfordshire, LU1 3JU, England, UK; 5 Postgraduate Medical Institute, University of Hull, Daisy Building, Castle Hill Hospital, Castle Road, Cottingham, East Yorkshire, HU16 5JQ, England, UK

**Keywords:** Chronic Fatigue Syndrome/Myalgic Encephalomyelitis, thematic analysis, social support, experiences, recognition, social welfare

## Abstract

**Background:**

Needs-based resource allocation is fundamental to equitable care provision, which can meet the often-complex, fluctuating needs of people with Chronic Fatigue Syndrome/Myalgic Encephalomyelitis (CFS/ME). This has posed challenges both for those providing and those seeking support providers, in building shared understanding of the condition and of actions to address it. This qualitative study reports on needs for equity in health and social care expressed by adults living with CFS/ME.

**Methods:**

The participants were 35 adults with CFS/ME in England, purposively selected to provide variation in clinical presentations, social backgrounds and illness experiences. Accounts of experienced needs and needs-related encounters with health and social services were obtained through a focus group (n = 6) and semi-structured interviews (n = 35). These were transcribed and needs related topics identified through data-led thematic analysis.

**Findings:**

Participants emphasised needs for personalised, timely and sustained support to alleviate CFS/ME impacts and regain life control, in three thematic areas: (1) Illness symptoms, functional limitations and illness management; (2) practical support and social care; (3) financial support. Access of people with CFS/ME to support from health and social services was seen to be constrained by barriers stemming from social, cultural, organisational and professional norms and practices, further heightened for disadvantaged groups including some ethnic minorities. These reduced opportunities for their illness to be explained or associated functional limitations and social disadvantages to be addressed through social support. Participants sought more understanding of bio-psycho-social aspects of CFS/ME, of felt needs of people with CFS/ME and of human rights and disability rights, for providing person-centred, equitable care.

**Conclusions:**

Changes in attitudes of health practitioners, policy makers and general public and more flexibly organised health and social care provision are needed to address equity issues in support needs expressed by people with CFS/ME, to be underpinned by research-based knowledge and communication, for public and professional education. Policy development should include shared decision-making and coordinated action across organizations working for people with CFS/ME, human rights and disadvantaged groups. Experiences of people with CFS/ME can usefully inform an understanding of equity in their health and social care.

## Background

Providing equitable care for people with Chronic Fatigue Syndrome/Myalgic Encephalomyelitis, commonly known as CFS/ME, has posed particular challenges for health and social services. The concept of health equity takes a critical perspective on determinants of health. As Marmot (2008) [1] argues, differences in health between groups of people that could be prevented by reasonable action are unfair. A health equity perspective requires inequities in the distribution of resources that shape health outcomes to be questioned. This article reports a qualitative research study that examines challenges for equitable access of people with CFS/ME to appropriate health and social services in England where there are state-provided services. Access to these, however, is linked to, and often constrained by official recognition of health conditions. Where, as with CFS/ME, these are difficult to diagnose and to recognise as genuine illnesses, inequalities of access arise to health and to health-relevant social care.

CFS/ME is a serious problem, which in other countries has been shown to affect up to 0.4% of the population [2]. It may thus affect up to a quarter of a million people in Britain. However, exact numbers for the UK are not yet known, due to a lack of empirical population evidence. It is up to three times more common in women than in men, and affects all classes and social groups [3]. Up to 25% of people may at any one time be severely ill (housebound or bedbound) [4]. Little is known about prognosis [5], except that the illness may last for many years, with complete recovery unusual, and with a worse prognosis in those severely ill [6,7]. Inappropriate care in early stages of illness appears to be associated with the development of severe disease and a poorer prognosis [8].

Quality of life is linked to a broad range of human experiences related to overall well-being [9]. These are influenced not only by morbidity and functional status, but a wide range of domains like health promotion, personal care, independent living, family roles, work,, leisure and material well-being [10]. Social exclusion implies restriction in participating in these domains. Considerable evidence suggests the needs of people with CFS/ME for illness management and social inclusion are often unmet and lead to a lower quality of life [11] for themselves and their familiesThe disease is poorly recognized by health and social professionals and the general public. It affects neurological, gastrointestinal, cardiovascular and muscular systems, with a variety of symptoms, including severe muscular pain and physical and mental fatigue, which may fade, reappear or worsen over time, varying between individuals [12]. About 25 per cent of those with confirmed diagnoses become housebound or bedridden and most experience adverse effects on functional status [13,14]. Lack of consensus about the nature of the illness, case definition and even terms such as CFS or ME contribute to uncertainty on clinical guidance and epidemiological information.

Diagnosis can be complex; a study of patients having fatigue lasting more than six months and seen by GPs, using the 'Centres for Disease Control' criteria, [15] found one third had CFS/ME [16]. A lack of specific diagnostic tests or physical signs restricts the process of diagnosis to clinical history-taking, examination and exclusion of other diseases. Delay in establishing a diagnosis of CFS/ME delays access to many types of support. It is often only diagnosed after long term suffering, sometimes over ten years after symptoms began [4]. Lack of knowledge and inadequate communication between patients and professionals has often been barriers to diagnosis and care as a CFS/ME diagnosis may rely on patients accurately relating histories of their health problems. **T**here is no specific treatment, and those offered mostly focus on alleviating impairments and improving functionality.

Some doctors lack acceptance and knowledge of CFS/ME [17-21]. Group discussions with 46 GPs in England [22] showed that they tended to negatively stereotype patients with CFS/ME as having anti-social characteristics, including failing to conform to the work ethic or to acceptable sick roles, in conflict with their doctors about the causes and management of their illness, so raising barriers to effective care. A recent survey found that while 72% of GPs accepted CFS/ME as a recognised clinical illness, 48% lacked confidence in diagnosing and 41% in treating it [23]. GPs who recognized CFS/ME as a genuine condition [24] saw more patients with the disease or knew someone socially who had it, had more positive attitudes towards patients with CFS/ME. Where their GPs do not acknowledge or do not know about living with CFS/ME, this may lead to people with CFS/ME having higher levels of depression, anxiety and social exclusion [25]. Health professionals negative attitudes may be explained by problems with case definition and contested labels such as CFS, making it easier to aggregate a heterogeneous mix of patients as a single group, or to view the illness wholly as a psychological condition. Physicians' discomfort with uncertain diagnosis and management protocols [26], increasing pressures to rapidly diagnose and treat, and communication problems between people with CFS/ME and care professionals, may exclude patients from available treatment and social benefits and lead them to seek complementary forms of treatment [27].

Evidence based clinical guidelines for CFS/ME recommend a holistic management based on biological and clinical research, expert opinions and social research on users' views on their illness and needs. The UK guidelines point to insufficient evidence about the felt needs of people with CFS/ME [28].

Exclusion from welfare benefits also follows from a lack of acknowledgement of the impact of CFS/ME on functional ability. Thanawal and Taylor [29] found that lack of financial resources and of knowledge of service availability were the two most frequently reported barriers to service use in a sample of 47 adults with CFS. People with CFS/ME are more likely than people with other diseases to have their claims for disability benefits denied by occupational doctors, but to receive such benefits after legal appeal [30].

Social exclusion relates to lack of participation in domains related to wellbeing, and may be particularly severe if discrimination is due, not only to having CFS/ME, but also to other factors which make participation difficult. These include age, gender, sexual orientation, ethnic background, poverty and local facilities.

There is evidence of widespread social inequalities in the health of the CFS/ME community [23]. Social exclusion from healthcare may be even greater for the non-White population and for women [31,32]. Community-based studies related to CFS/ME have found higher levels of this illness in non-White populations [33] but lower levels of shared recognition of it [34], other types of social inequalities in health which may be associated with poor prognosis [35].

This indicates the need for studies specifically addressing the needs of people more likely to be disadvantaged in their access to adequate care, such as those living in low socio-economic conditions and from ethnic minority groups. The study reported here therefore aimed to investigate the impact of CFS/ME on people from varied social backgrounds, including those from ethnic minorities, and what challenges may be posed to health care practitioners in providing appropriate and equitable care for this condition.

This study is part of a National Observatory of people with CFS/ME in England. This aims to produce and to facilitate epidemiological and social research, in response to the needs of these people so as to fill a major gap in the evidence of the occurrence and the impact of this disease.

The research to be reported here provides analytic findings from the views of people with CFS/ME themselves about support they need from health and social services, identifying major sources of inequities in healthcare, and indicating strategies needed for their needs to be equitably provided.

## Methods

The study design was a qualitative inquiry using in-depth, semi-structured interviews and thematic analysis.

### Participants and procedures

The participants were 35 adults (18 years and older) with CFS/ME in England. Purposive sampling procedures aimed to recruit participants representing maximum variation in the experience of living with CFS/ME, and of opinions about what is needed from health and social services across England, drawing on diverse ethnic and socio-economic backgrounds, illness severity and duration.

Recruitment began in November 2006, after ethical approval (MREC 06/MRE02/58), and ended in March 2008. Initially, the researchers (MD and CS) contacted relevant support groups, community organisations and centres, practitioners, and media to publicise the CFS/ME Observatory and this study across England.

People living with CFS/ME who approached the research team as willing to take part were posted general information on the CFS/ME Observatory and detailed information about the study aims and procedures,, a brief questionnaire of illness experience and socio-demographic details, and a stamped addressed envelope to return a slip confirming willingness to take part. From the pool of 52 potential participants who returned this slip, the researchers successively selected participants purposively, until the sample captured a diverse range of illness severity, duration, social variation (age, gender, ethnic background and socio-economic conditions)and year of diagnosis **(**Table 1). Saturation in relation to types of illness experiences and needs were also seen to have emerged in the interview dataset.

**Table 1:** Social, demographic and illness characteristics of the study sample

Participants	Age group (gender)	Ethnicity	Highest level of education	Employment	Disease duration^1^
P1	41 to 55 (female)	White British	university	retired	7 ≤
P2	41 to 55 (female)	White British	university	unemployed	7 ≤
P3	26 to 40 (female)	White British	university	self-employed	1-3
P4	56≤ (female)	White British	university	employed	7 ≤
P5	41 to 55 (female)	White British	college	unemployed	7 ≤
P6	26 to 40 (female)	White British	secondary school	unemployed	4-6
P7	26 to 40 (female)	White British	college	unemployed	7 ≤
P8	18 to 25 (female)	White British	college	student	4-6
P9	56 ≤ (male)	White British	university	unemployed	7 ≤
P10	18 to 25 (female)	White British	college	student	4-6
P11	41 to 55 (female)	White British	university	unemployed	7 ≤
P12	18 to 25 (female)	White British	secondary school	unemployed	1-3
P13	56 ≤ (male)	White British	university	employed	7 ≤
P14	26 to 40 (female)	White British	college	sick leave	7 ≤
P15	56 ≤ (female)	White British	secondary school	retired	7 ≤
P16	41 to 55 (female)	White British	university	unemployed	4-6
P17	≤ 56 (male)	White British	university	unemployed	4-6
P18^e^	41 to 55 (female)	German-Jewish	university	employed	7 ≤
P19	41 to 55 (female)	White British	college	employed	7 ≤
P20	41 to 55 (male)	White British	university	retired	7 ≤
P21^e^	26 to 40 (female)	Chinese	university	employed	7 ≤
P22	56 ≤ (male)	White British	university	retired/self-employed	7 ≤
P23	56 ≤ (male)	White British	secondary school	retired self-employed	7 ≤
P24	41 to 55 (female)	White British	university	employed	7 ≤
P25	41 to 55 (male)	White British	secondary school	retired self-employed	7 ≤
P26	41 to 55 (female)	White British	university	retired	7 ≤
P27^e^	41 to 55 (female)	African	university	sick leave	7 ≤
P28^e^	41 to 55 (female)	White-black African	secondary school	unemployed	7 ≤
P29^e^	41 to 55 (female)	White-black Caribbean	secondary school	unemployed	4-6
P30^e^	26 to 40 (female)	White & Asian	university	sick leave	1-3
P31	56 ≤ (female)	White British	college	Housewife	7 ≤
P32	26 to 40 (male)	White British	secondary school	retired	7 ≤
P33^e^	41 to 55 (female)	Portuguese	secondary school	unemployed	7 ≤
P34^e^	18 to 25 (female)	Chinese & White	college	student	7 ≤
P35	26 to 40 (female)	White British	university	unemployed	7 ≤

Six of the 35 participants were purposively selected (to include a diverse range of illness severity, duration and social variation) for both an initial focus group discussion as well as later one-to-one interviews with a researcher. The other 29 were invited to take part in one-to one interviews only.

### Description of participants

Table 1 lists characteristics of the 35 participants, including age, gender, education, employment, ethnicity, illness duration and time since diagnosis.

### Data collection

The research data elicited through focus group discussion and the one-to-one interviews were descriptions of experiences, beliefs and feelings about living with CFS/ME and being managed within health and social care services in England. These were tape-recorded and transcribed *verbatim *with the written informed consent of all participants.

***The focus group ***with six people with CFS/ME was used to identify the main themes and issues to be explored more deeply in the subsequent interviews. The group's dynamic facilitated participants to consider their own and others' views. It took place in a quiet room and lasted for two hours, with a break for refreshment and rest. The group was conducted by a researcher (MM), while another researcher (JL) supported the group dynamics, observed and took notes to facilitate later analysis. The discussion was managed as a conversation,, encouraging participants to tell their own stories to help articulate their ideas about the experience of living with CFS/ME. Three broad areas of enquiry reflected in guide questions were used as starting points to encourage story-telling and discussion to facilitate the emergence of story line narratives within these areas: (a) becoming ill and being diagnosed; (b) the impact of living with CFS/ME; and (c) self-management and being managed within health and social care services. Story-telling allowed themes to emerge, without being fixed to a set research agenda. The sequence and wording of questions were decided in the course of the discussion to respond to participants' preferences and conversational styles.

***One-to-one semi-structured interviews ***of about 45 minutes (up to a maximum of 3 interviews per participant, 45 interviews in total) were conducted with the 35 participants by a researcher (JL) at the participant's home or another place convenient for them. The interviews were managed as a conversation guided by questions informed by analysis of the focus group data.

### Data analysis

The focus group and interview data having been transcribed verbatim, thematic analysis was used, as a systematic method of identifying themes or patterns within the data [36]. This method is accessible to an educated public, and recommended for research aimed at informing policy and service development [37]. Inductive thematic analysis was carried out on both the focus group and interview datasets.

The focus group data transcript was analysed by four researchers (JL, MD, AK and FP), who together identified the main storylines and emerging thematic areas of support needs, and then adapted the question guides for the one-to-one interviews.

The one-to-one interview transcripts were analysed by five researchers (JL, MD, AK, SK and FP) drawing on the inductive approach suggested by Miles and Huberman [38]. They first independently read and re-read the transcripts to identify and extract words and text sections which appeared to describe experiences of living with CFS/ME and encountering health and social services. They independently selected, focused and condensed the data in tabulated written notes, with codes such as 'not believed', 'understanding from employers helpful' and 'coping strategies'. Each code was linked in the table to text 'chunks' [38] and pre-analytic remarks together used to establish a preliminary code system which yielded many themes detailing experience of symptoms and their effects on their lives, relationships and changes over time related to the responses and support they received from health professionals, family and others. Following the initial development of the preliminary coding system, three researchers (JL, AK, SK) met to compare the reliability of codes and to agree the developed coding scheme. Before comparative subject analyses were carried out, these researchers developed new codes emerging during further individual analysis which included a richer variety of experiences of living CFS/ME and services and to ensure data saturation. Finally, a wider group of researchers (JL, AK, SK, L'OD, FP) drew conclusions for the whole dataset by identifying themes or patterns, contrasts, clarifying relationships and building an interpretative understanding from the set of narratives. The codes and themes identified emerged from participants' own issues over the whole data set, without reference to themes identified in previous research.

Steps to enhance trustworthiness aimed to ensure that project findings accurately represented participants' viewpoints [39]. Different researchers independently analyzed the data, and then checked understandings with each other (researcher triangulation, including authors from diverse professional and academic backgrounds in analysis and reporting, using a variety of research methods); individual and focus group interviews and observation to gather data (methodological triangulation); comparing the issues arising from this project with literature (theoretical triangulation); sharing the study results with participants to check whether their expressed main views were included, to be amended to take account of their response (respondent validation); review of the draft report by the steering group and reference group of the CFS/ME Observatory, with members of the CFS/ME community and other stakeholders to also inform the report (member checking); dissemination events to share key findings with CFS/ME community members to check their perceived relevance (member validation).

Participant narratives consistently highlighted how CFS/ME is a complex, distressing, problematically-perceived and debilitating condition with a major impact on quality of life.

## Results

The interpretative process helped to organize the narratives of equity in support needs into three interrelated thematic areas of perceived need for equity. These are summarized here as:

(1) Illness symptoms, functional limitations and illness management;

(2) Practical support and social care;

(3) Financial support.

### (1) Illness symptoms, functional limitations and illness management

Participants all reported that before becoming ill with CFS/ME they had had an active family, social and productive life as housewives, parents, offspring, friends, students, researchers, professionals, manual or clerical workers. They reported illness-related experiences of a wide range of distressing and debilitating impairments and functional limitations, varying greatly in combination and intensity between participants and over time. Seeking support for managing their health challenges characteristically involved them in lengthy and problematic processes, reflected in the sub-themes which follow, of: gaining recognition of *their symptoms, impairments and limitations *as illness; *achieving a diagnosis*; and gaining appropriate health services support to *manage their illness*.

*Experienced symptoms and impairments *commonly included severe fatigue experienced as highly disproportionate to the activities preceding them, not relieved by rest, usually accompanied by cognitive impairments such as memory and concentration difficulties. Pain was another major and common symptom, as well as sleep problems, such as overwhelming drowsiness alternating with frantic sleeplessness. Other distressing symptoms reported by some participants were: gastro-intestinal problems,, sensory and chemical hypersensitivities,, tachycardia, dizziness on moving, and frequent infections.

*Experienced limitations on activities *were also seen to vary greatly between individuals and between differing periods of the same individuals' experience with this condition. These ranged from being able to maintain activities of daily living, employment and communication at the cost of much effort and restriction in other activities, to major limitations, being wheel-chair-, house- or bed-bound with multiple hypersensitivities and pain.

Participants revealed how they were facing distinctive illness-related barriers, in gaining recognition of their illness. To manage this complex illness required people to gain access to appropriate health expertise which, in turn, could affect their likelihood of gaining family and wider social support . However, their encounters with health professionals were reported as often problematic in ways which both delayed or reduced access to such support and greatly exacerbated emotional pressures.

There were reiterated experiences of not being listened to by healthcare practitioners, as one young Asian woman commented:

*'...actually what she said to me.' I'm the doctor I know best....'. You see, when they say that to you, when they are not really listening to you, that's frustrating' *(P30 White-Asian female, educated to university level)

This often posed particular problems in the earliest stages of the condition. Nearly all participants, from both white and non-White groups spoke of their illness not being taken seriously by GPs, with individual symptoms being dismissed, perhaps as 'a virus' or as a common cold. Many participants experienced this as a profound lack of acknowledgement:

*'(...). when I was telling them it was like this (...). They said. oh no, just shhh, we'll give you anti depressants so you can just go, go be quiet somewhere and it's just like, they weren't listening' *(P34 Chinese-White female college student).

This was echoed by most non-White participants as in:

*'I don't think I was treated seriously, they didn't take on board what I was saying and my symptoms'(P29 White-Black Caribbean female, educated to secondary school level)*.

Such difficulties have a particularly marked impact on equity, as GPs are frequently required to act as gatekeepers to other health services, or to social resources, leading to treatment delays. As one person commented:

*'She was often very reluctant to provide a formal letter and I always dreaded having to go and see her when I needed another letter because I felt I was always having to justify my illness and justify myself and I felt that she thought I was making an excuse.' *(P2 White British female, educated to university level).

Such recognition was also central to gaining most forms of assistance from health or social care needed to enable them to participate in both public and private social life, including work, education, leisure and family life. While clearly an issue for the majority of participants challenging such lack of recognition could be especially difficult for people from ethnic minority groups in which such illnesses were less commonly identified as self-recognition and belief of the symptoms and experiences was also problematic.

*'I never heard of any black person, or any other ethnic minority who's got this illness (...) I just thought maybe, you know, this is a white middle class illness' *(P27 African woman, educated to university level).

This was reflected in participants across all ethnic groups wanting health care practitioners to have the time to help the patient feel *'empowered' *(P24), and '*believed*' (P1, P34), to increase their sense of inclusion and acknowledgement.

*Achieving a diagnosis *was seen as the crucial milestone for most participants (P1, P3, P4, P5, P7, P9, P11, P14, P32, P34). Where this led to advice from doctors and other health care professionals with particular knowledge of CFS/ME, this was almost invariably a positive experience. For example, one participant commented on his unusual luck in gaining a prompt GP diagnosis, leading to coordinated care and support from his manager, which allowed him to work part-time within his capabilities and to gain sick leave and retirement as the illness progressed (P32). Most participants, however, found doctors saying they could not help, resulting in their feeling abandoned to fight the problem by themselves:

*'I don't think I have seen anybody for about two years now, and I haven't had my medication reviewed properly during that time or any specific help from a doctor since. I've been pretty desperate really, because (...) I want to move forward. (...) a lot of people don't believe me while the GP doesn't seem to believe that these symptoms exist.' *(P1 White British female, educated to university level).

Even when bed-bound, participants encountered unsupportive attitudes from health professionals which greatly undermined their chances of wider belief and support:

*'I was more or less bed bound for a couple of years, (...)and meanwhile the doctors were telling me there was nothing wrong with me. So I was under pressure from employers, family and everyone else to stop imagining that I was ill, and to get out of bed and get on with my life.'*(P9 White British male, educated to university level).

Disagreements over diagnoses and over-attention to psychological symptoms could lead to inappropriate treatments which paradoxically contributed to deterioration in emotional well-being:

*' (...)the only stress I'm suffering now is the stress of being ill, you know the frustration of it. I wanted to get back to work; I was anxious about my classes, so the doctor kept putting on my medical certificate chronic anxiety state.' *(P20 White British female, educated to university level)

While these participants felt that the health care system should explore useful interventions, they more often encountered oppositional health services responses. Some therefore decided to use a private or alternative health services as a way of getting diagnosis or help, often exacerbating stress, uncertainty and financial pressures:

'I was lying on the floor in pain a lot of the time and all I had was the doctor just giving me pain killers. (...)I said I wasn't suffering from stress. I am stressed because you're not doing anything about it (....). Then I had some private medical insurance. (...I saw so many different people and I saw this osteopath that I paid privately. He thought I had ME so I paid privately to go and see Dr ** (NHS consultant) (...) so I could get a diagnosis.' (P16 White British female, educated to university level).

*Illness management*. Participants suffered from lack of control over choices of treatment for managing their illness, which they saw as due to both lack of resources in the National Health and social systems and relative lack of recognition or value given to their own experience with the illness. Where participants' and GPs' views differed on appropriate treatment, typically around graded exercises or antidepressants, a refusal to take antidepressant medication was often interpreted by the GP as a refusal of treatment. Participants desperate for relief of feelings of pain or illness reported finding treatments such as massage, osteopathy, dietary advice and acupuncture helpful, and it caused ongoing frustration that such interventions were not funded by either the NHS or by private health insurance for CFS/ME (P12, P16, P21 P27, P28, P29, P34). Citations show these as especially likely to be mentioned by participants from ethnic minorities.

People who had hospital care described their need for designated wards for CFS/ME, with environments adapted to their needs, as in keeping light and noise levels low (P10). Some participants highlighted the limited time for consultations as a barrier to appropriate care provision and another reason for seeking support outside the NHS. For example, one Asian woman (P30) explained that her NHS acupuncturist limited treatment to only three needles, whilst the private service she eventually attended but could only afford for a limited time allowed the acupuncturist time for enough needles for pain relief and to discuss her situation.

People from minority ethnic backgrounds reported particular difficulties in accessing health and social care support systems, experiencing more stigmatisation and stereotyped responses that did not fit their health needs. For example a Caribbean woman said she was not taken seriously because of her ethnicity, with all symptoms interpreted as psychiatric in origin;

*'They sent a psychiatrist round to ask me if I was having problems and you've got so many children and you're separated from your husband and I was saying no, I've been separated from my husband for years why would it just come now' *(P29 White-Black Caribbean female, educated to secondary school level).

They expressed a need for workers from the same culture who could relate to their background. An African woman (P27) observed that *'pain in Black people is stigmatised and you see more black people in mental health than other people...' *going on to report being given injections roughly, within a system where attitudes were often unsympathetic to both her illness and her ethnic background.

Some non-White participants therefore experienced ethnic group-specific lack of understanding of their condition and situation in trying to access a diagnosis. Just as the quality of social relationships was seen to be closely bound up with processes of accessing health services, access to social care and support was seen as vital for managing their lives and for practical support.

### (2) Practical support and social care

In relation to the theme of practical support and social care, sub-themes detailed the importance participants' attached to securing: *practical support *to avoid escalating the illness, and associated financial problems; *social care *given and received in gaining control over their lives; and so to secure *social and cultural inclusion *to optimise wellbeing within the limits of their illness.

*Practical support *for personal care, family roles, independent living and support for carers was invariably seen as extremely important for people with moderate to severe illness. Many participants described needing help with all personal and domestic tasks: with moving around the house, getting out of bed and from chairs, washing and dressing, feeding and self-care, running a home, including meals preparation, shopping or cleaning (P5, P6, P9, P11, P12, P35 P10, P27), and how these intensified with childcare:

'*When you have children it's not just the meals, it's the homework, it's the washing and ironing, you know, it's the full Monty.' *(P16 White British female, educated to university level).

One person described teaching her five year old daughter to phone for help and to make herself a drink in case she found her mother unable to move. She commented that in appointments GPs never asked her about how the home was being run (P26). Participants described other family members having to cover such family responsibilities, as with a young mother, a single parent, whose own mother covered this role (P35), whose ten year old child was doing the housework (P16) and, refused practical home social care support even when, as with a Chinese participant her older mother-carer, became ill, this led to an eleven year old friend cooking and doing housework for both of them (P21).

While participants were aware of the need for a balanced diet to maximise their limited health and energy, several described having to go without food when they were physically unable to prepare it:

*'I needed complete rest and somebody to do the shopping, to do the cooking and (...) sometimes I needed weight builders' food because I didn't even have the energy to hold a fork so I needed complete care really.' *(P9 White British male, educated to university level).

*Social care*, both given and receivedcould be seen as an issue of paramount importance for most participants' life priorities which often included sustaining their own roles as family caregivers. A participant who was mostly bedbound with hypersensitivities to sound, light, touch and chemical products said that, to support an independent life, she would need trained carers and highly specific home adaptations (P35). These required improved access to social care.

They highlighted how lack of access to social care and practical support was exacerbated when health practitioners would not recognise their illness, making a profound impact on their ability to carry out their family caregiving roles, particularly as parents. Participants described how 'most people with CFS/ME don't get social care' raising serious questions about how people might go on and manage their lives with the debilitating symptoms they reported. Nonetheless, until a diagnosis was gained, social services often could not even assess their needs (P5). Without social care support, often partners, parents and sometimes children had to become carers. One, not unusual, example was described:

'*On my bad days I didn't have any help. I've got a four and a half year old and a six year old (who) couldn't have gone to school. She would have been here on her own with me. (...) ...I couldn't do anything physical at all. (...) I had to tell her how to programme the phone in case I collapse.' *(P3 White British female, educated to university level).

Inevitably minimal access to other forms of support also had an impact on carers, as P1 explained:

*'My husband has been ill at times and I'm sure it's just the stress because he never stops, he has to do all the shopping, (...). He has to do everything. Without him I would just never survive on my own. We paid for a cleaner to come in for three hours on a Wednesday morning (...) as my husband has to work and we get no social help or no disabled badges or anything.' *(P1 White British female, educated to university level).

A participant whose mother had given up work to care for her described the severe strain on family life in providing care for two adult offspring with CFS/ME and her grandchildren (P8). A male participant recounted the collapse of his relationship with his partner, under such strain (P13).

*Maintaining *s*ocial and cultural inclusion*. Participants' daily living was therefore greatly affected, both directly and indirectly, by CFS/ME, from inequities in access to health and, relatedly, social support and social inclusion. Participants recognised that CFS/ME was a chronic condition, which could have a catastrophic impact on their lives, with no ready 'cures'. However, even if treatments were not available, they still needed services to help reduce the impact of the condition, to make life more bearable and to reduce exclusion:

*'(...) if somebody cannot use their legs properly you should give them a wheelchair' *(P20 White British female, educated to university level).

Instead many underlined their heightened experiences of social isolation and dependency.

*'I'm house trapped and my husband does most things' *(P15) linking these to extensive needs for practical support: *'I couldn't lift a cup or get downstairs by myself. I couldn't get out of the house.' *(P6, White British, educated to secondary school level). Such perceptions of exclusion were heightened for some non-White participants:

*'I know if I happened to be a white person the story will be different. They'd probably be more sympathetic, yes it does affect whether you're black or white, Asian' *(P27 African woman, educated to university level).

Where people could not find alternative ways of getting support for practical tasks, the home environment, where they had to spend most of their time, becoming, not their refuge, but a further source of stressful experiences of deteriorating well-being. Such issues pointed up further inequities in the financial burden arising from the health and social demands of living with this condition.

### (3) Financial support

Financial support was consistently identified as crucial for illness- and life-management, and to maintain education, social relationships and material well-being. Yet all participants described many financial constraints arising in the absence of other forms of support to live with CFS/ME. There were primary consequences of impoverishment, but then secondary consequences for social standing, relationships and future entitlements. Limited incomes imposed hard choices about what money would be spent on, which debts to run up. These are presented in sub-themes concerning: *private income, savings and insurance*; *accessing welfare benefits *and; *accessing employment*.

*Private income, savings and insurance*. As well as finding their ability to gain income reduced, participants faced additional expenses with CFS/ME. Feeling 'written off' by state health provision, many described paying for relatively expensive treatments to manage pain, or diets to optimise their wellbeing (P23, P34). Some participants had benefited from resources such as medical- or employment-related insurance (P16, P13, P11, P25), some using these to get specialist assessment and diagnosis more speedily. One participant found that private insurance quickly stopped paying for treatments for CFS/ME, arguing that they were not known to be effective (P13). Another refused to pay out on a critical insurance policy when the participant had to give up work through CFS/ME, seemingly just as poorly acknowledged in the private sector. A few participants had managed by living on savings or renting out property, but saw their financial situation drastically reduced by the impact of CFS/ME (P13, P25). For participants affected from school age, their parent(s) may have had to reduce or to stop working altogether to care for them, affecting the whole family's income (P30, P35).

For people with no private insurance or savings to fall back on, and unable to work, the main way forward was to apply for state benefits.

*Accessing welfare benefits*. Participants described the frustrations of attempting to apply for welfare benefits while affected by CFS/ME.

*'I have recently been running around even though I've got problems, because of the DLA [Disabled Living Allowance], to try and get everybody to speak to each other' *(P28 female White-black African)

The nature of CFS/ME symptoms, with extreme fatigue, pain and cognitive impairment, meant that complicated forms having to be completed even to start the process, were especially daunting. Where benefits assessments were based on questions about function, people felt acutely that their limitations, which often varied greatly over a time period, could not be adequately represented and recognised.

'*the way they ask the questions and (...) when you answer, can you prepare a meal, (...) because you say "occasionally I can" they disregard you straight away.' *(P33 White European female, educated to secondary school level).

Many participants described the clear official lack of understanding of the variable nature of the condition, and that despite occasionally being able to carry out certain activities, the overall impact on functioning was severely limiting. One person insisted on an assessing doctor spending several hours with her to appreciate the impact on fatigue and function of carrying out tasks (P10). Participants frequently found the benefits system complicated and confusing, something to 'fight' rather than a source of support. Not being able to predict what they were entitled to, people found out in a hit-and-miss way, making arduous applications for benefits that were then often refused. While some from both White and non-White groups, pursued appeals, with help from welfare rights services (P10, P14, P7, P35, P32, P33, P23, P29, P34, P1, P16), several did not feel well enough to pursue such battles (P5, P9, P30, P29, P3). Many participants, especially from non-White groups, expressed their needs for much more information on entitlement to help focus their applications on attainable benefits (P3, P5, P7, P16, P19, P20, P21, P23, P27, P28, P32, P34, P35). However, most participants saw the inflexibility of such forms as "*inappropriate for assessing the level of disability in ME term: they don't give scope to express the worsening of symptoms restricting activities" *(*P35*, White female, educated to university level).

Some described the irony of only being able to appeal and succeed in gaining benefit when they were better. One Chinese woman, advised by welfare rights workers to make claims on the basis of symptoms such as anxiety and depression rather than CFS/ME, saw her diagnosis of CFS/ME as impossible for the system to recognise (P21).

Some participants, unable to claim any benefits, whether through lack of support from benefits staff or their current financial situation, experienced profound effects on their lives, being bereft of resources, and enforced total dependence on family or partner.

'*Me never having any income of my own really became a really big issue with me and my partner and that became the stick he beat me with metaphorically (...) and the same with your social standing with your peers (...) if you are not getting sickness benefit you are seen as a sort of a drain.' *(P5 White British female, educated to college level).

Where not getting benefit and feeling too unwell to sign on for unemployment benefit, people could then experience a lack of acknowledged civil status and, without being credited for National Insurance payments, worries about the implications for later claiming pensions (P27, P5).

Where benefits were successfully secured, these were always perceived as helpful. Two participants were able to use their Incapacity Benefit to regain social engagement by supporting themselves to study part time (P2, P16). A few gained Disability Living Allowance (P6, P15, P32). One person described being helped by Social Services staff to apply for 'direct payments', to employ somebody to carry out personal and domestic tasks, help with managing finances and, importantly, leaving the house for social contact (P32).

Some people could use state benefits, private insurance or savings to pay for different forms of practical help, including: employing an *au pair *for help with domestic tasks and childcare, (P24, P6), and employing a 'personal assistant', not just for physical domestic tasks but with home administration, like managing finances (P26).

*Accessing employment: *For people beginning to recover and wanting to increase their activities gradually to include limited part time work, income support was seen as too inflexible to allow this. The benefit stopped if people started work, even when they could not work sufficient hours to earn enough money to support themselves (P15, P19, P24). Their condition posed other dilemmas; to obtain essential benefits they needed to represent themselves as very impaired, yet in attempting to move back into employment or education, they had to represent themselves as minimally affected (P16, P18, P19, P12). Pressures from both rigid benefits systems and inflexible routines were found often to impede their recovery and continued employment or social participation.

The specific inequities participants faced were therefore closely linked to the attitudes, knowledge and behaviours of practitioners in health and social services and the lack of appropriately responsive, personalised services to meet their complex needs for health and other practical support, including means to manage financial demands. The interactions between these factors frequently increased rather than reduced barriers, so creating a socially unsupportive system experienced as unresponsive and inequitable. However, while non-White groups were seen to highlight their difficulties in securing financial support (as for social and practical support) they mostly did not draw further, explicit links with their ethnicity. Most participants called for more research to help evidence the treatment, management and social support for CFS/ME, together with more training for health professionals (P2, P15, P15, P18, P20, P21, P22, P25, P26, P35).

## Discussion

This study investigated the expressed needs for health and social care of CFS/ME of people from varied social backgrounds, including those from ethnic minorities, and the challenges posed to health care professionals in providing appropriate and equitable care for this condition. As Marmot [1] argues, differences in health between groups of people, that could be prevented by reasonable action, are unfair and analysing these participants' accounts reveals devastating and exclusionary experiences stemming from the limited understanding and responses of others to their condition. Their accounts offer detailed reasons for their low quality of life as found by other studies such as Anderson and Westwing [40]. However, these findings ground these in wider experiences of cumulative exclusions from previous life activities and relationships and also from the social and health professional support needed to help regain health or at least manage their lives with some respect. Our results point up the pressures imposed in seeking to live acceptably with the illness, the vital role of social and professional acceptance, knowledge and support and, where these are absent, the systematic inequities in health and social care that follow.

The underlying structure of their expressed needs is summarised in Table 2 and discussed in two parts, (1) the barriers to equity in meeting support needs in health and social care, and (2) the priority policies and strategies for equity suggested by participants' accounts and considered in the context of other findings.

**Table 2:** Support needs and priority strategies for equity in health and social care, and risk factors for increased inequity from participants' experiences of living with CFS/ME

**Priority policies and strategies from experiences of CFS/ME**	**Public and professional relevant knowledge**
	CFS/ME
	Human and disability needs and rights
	Needs-based care
	Social inclusion and health and social equity
	
	**Understanding of needs of people with CFS/ME**
	Acceptance
	Illness recognition
	Proactive attitudes
	↑ *Supporting*
	**Values and practices**
**Support needs for equity in health and social care**	User power and control
	Needs-based choice
	Inclusive
	Non-stereotyping
	
	**Policies and systems**
	Responsive and flexible
	Integrated and coordinated
	
	**Health and social services**
	Supportive
	Personalised
	Mutually respectful
	Timely
	Continuous
	
	**Quality of life**
	Social inclusion
	Wellbeing optimised
	Purposeful living sustained
	Illness managed
	Social support
	
	***↓ ****Undermined*
	Illness/disease status not recognised or legitimised
**Risk factors for increased inequity in CFS/ME experience**	Limited psychological/physical explanation for illness
	Age and gender stereotypes
	Ethnicity (soc exclusion, cultural boundaries, services access, stereotypes)
	Health and social benefits knowledge, accessibility, entitlement
	Citizenship (postcode lottery, national insurance entititlement)

### Barriers to equity in meeting support needs in health and social care

For these participants, making sense of the illness and gaining a diagnosis was seen to be crucial for self-management. These become vital where illness-related needs fluctuate requiring prompt and sensitive responses which include appropriate management within the health system and gaining support in other areas of life, as within networks of family and friends, education, work, social security and pensions. Although fulfilling these needs depends on the ability to communicate symptoms by the person with CFS/ME, family and friends, the key to gaining a diagnosis is seen to lie with the health professionals they encounter, crucially at the GP consultation stage. Participants described unhelpful successive meetings with health professionals which made them feel 'powerless', 'disbelieved' and 'isolated'; whilst in helpful meetings they referred to feeling 'relieved', 'trusted', 'confident'. Scepticism of health professionals, particularly GPs, in recognising that CFS/ME exists, still poses a problem. A survey in the UK, of 46 GPs' beliefs relating to CFS/ME, showed 44% as considering this illness condition not to exist [41]. Research findings since 1995, are not yet incorporated in health professionals' training [42].

Caring for people with CFS/ME can challenge health professionals. Negative attitudes toward GPs were expressed by almost all participants, as reported in other studies [43,44]. Similarly, GPs often perceive this patient group as difficult to deal with [24]. But GPs are also known to be important in helping people to understand and deal with this incapacitating illness [45]. While being believed seems crucial to building participants' trust in their relations with health professionals, medical training specifically encourages reliance on objective signs. Restrictions on time, specialist knowledge of CFS/ME and cultural attitudes including age, gender and ethnicity stereotyping may also contribute to uneven trust in treatment relationships.

Social cognition models applied to other chronic illnesses point to the roles of significant others where the mediating factors of support and understanding help in coping with chronic illness. To counter powerlessness, people with chronic illness are advised to attempt to pass on control to significant others, like their families, or sympathetic health professionals who can protect the person from stressors, by reducing the stigmatizing potential of the illness through increasing self-esteem [46,47], by providing practical support and facilitating access to care [3]. However, for these study participants sources of such social support were less common, their need for support was frequently disbelieved, their social and civic status diminished and their access to both health and social support consequently reduced. Participants therefore either remained overwhelmed, or turned to self-management activities, often forced to rely on their own knowledge and on scarce and dwindling resources.

These participants expressed clear awareness that their quality of life was seriously affected by inequities in care closely linked to attitudes, knowledge and behaviours of service practitioners, and to unfair policies, systems and services which were inadequate to meet their specific needs for health, other practical and financial support. Their consequent attempts to access services were frequently reported as exhausting, demoralising and isolating.

Insufficient expertise in dealing with people with complex and partially-explained needs, and lack of respect for human and disability rights lead to discriminatory norms and practices towards people with CFS/ME. Their situation was seen to socially disadvantage them, and also people connected with them, within an environment of prejudice and inadequate resources.

Participants explained how appropriate care could be provided through empathic, personalised, timely, holistic and coordinated support from health and social services. Guidelines for overall management of CFS/ME and for supporting people with long-term conditions and complex needs (3) point out that health professionals should act as advocates for their patients in negotiations with employers, educational institutions and social welfare organisations, and also in arranging part-time work or school alternatives as needed to enable them to participate equitably in both public and private social life, including health care, work, education, leisure, family life and social security. Participants' accounts describe devastating and isolating experiences, resulting from the scant understanding of their condition and individual and systemic disrespects to human and disability rights. Their stories affirmed the critical place of choice and control identified by Larun and Malterud [48] as contributing to whether people with CFS experienced physical activity as beneficial or harmful, and in developing suitable strategies to achieve positive energy-activity balance across life activities which facilitate inclusion.

**Figure 1:**
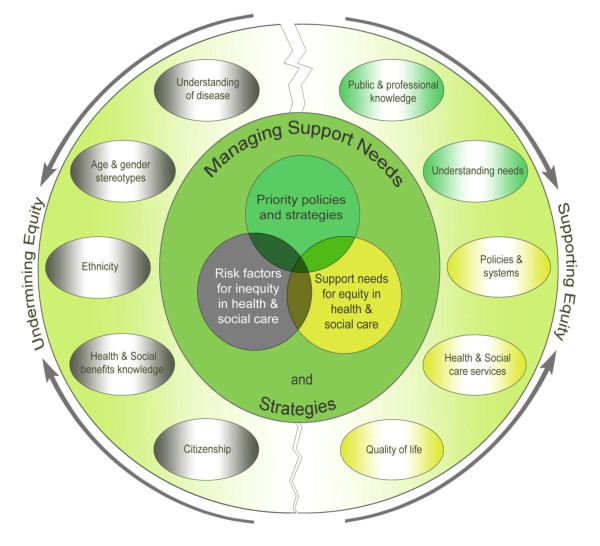
**Summary of dimensions of equity and inequity in managing support needs and strategies expressed by people with CFS/ME and factors undermining and supporting equity**.

However, the difficulties in overcoming barriers to access support appeared overwhelming for people unused to the complicated welfare support system, for those from minority backgrounds or in disadvantaged socio-economic conditions, and also when criteria for accessing support were too rigid to apply. The dimensions of equity and inequity and attitudes undermining and supporting them are set out in Figure 1. The interactions between these factors frequently heightened rather than ameliorated barriers to their well being.

Many people with CFS/ME are unable to work full-time, and those moderately to severely affected cannot work at all, so that financial difficulties often rapidly arise. Similarly, CFS/ME frequently disrupts educational studies. This study shows those participants, where state benefit was secured and who were willing or able to do some work, then faced losing their entitlement to social security benefit. Successful return to work or school, after a such a prolonged illness, requires a personalised and coordinated rehabilitation programme incorporating medical treatment, psychological support, occupational therapy and a more flexible policy towards the maintenance of state benefits.

The findings of health inequity may help explain previously reported widespread social inequalities for the CFS/ME community [23]. Social exclusion from healthcare was seen to be greater for the non-White population and for women [31,32]. While studies have found a higher ratio of White people with CFS/ME in tertiary care than in the local ethnic mix, studies outside the UK of community prevalence CFS/ME have found higher levels of CFS/ME within non-White populations [34,49,50]. Luthra and Wesseley [33] and Buchwald *et al*. [34] found people with CFS/ME from non-White populations as having less social support than those in White populations. Such associations in disparities in health and social access between ethnic minority groups and others in the UK are supported by wider reviews of evidence like that of Szczepura [51] which identified the clear need for health services to be responsive to the cultural needs and practices of patients if diverse populations are to have more equitable access to health care. In our study, ethnic minority respondents' experiences could be seen to mirror many aspects of those of White people with CFS/ME, but in some cases as undergoing additional exclusions, not always self- linked to ethnic groups. A recent CFS/ME Observatory epidemiological study of CFS/ME in primary care in England [52], found relatively lower proportions of non-White than White people diagnosed, suggesting that practices may under-diagnose CFS/ME in these groups. This complements evidence for adolescent girls from less deprived areas, that consulting behaviours may affect chances of gaining a CFS/ME diagnosis [53], However no studies have yet explored possible ethnic disadvantages linked to consulting practices relating to CFS/ME in primary care.

Other types of social inequalities in health within the CFS/ME community such as poorer education and employment have also been associated with poor health outcomes [35]. These raise the need for studies to address the needs of people with CFS/ME who seem likely to be at even greater disadvantage in their access, via consultation and help-seeking practices, to adequate care, such as those living in disadvantaged socio-economic conditions, and people from black and other minority groups.

If the current health and social system based on work status, conditional citizenship and similar criteria has been seen to restrict access to adequate care, as in this study, this suggests a human rights-based system may be needed to secure more equitable support for people with CFS/ME and similarly complex conditions.

### Priority policies and strategies suggested by participant experiences

The practical value and impact of available health and social services was seen to be constrained by experienced inequities in care, driven by lack of wider understanding of bio-psycho-social aspects of CFS/ME, dismissal of felt needs and experiences of people with CFS/ME, insufficient expertise in dealing with people with complex needs and overlooking human and disability rights.

Pro-active attitudes towards health and social equity should be backed up by producing and communicating knowledge and advocacy, based on further research, and through public and professional education. Expertise in CFS/ME and a multidisciplinary holistic approach to people with unexplained long term conditions and complex needs was seen to facilitate early diagnosis and satisfaction with care. Health professionals, particularly GPs, have a direct role in diagnosis, illness management and emotional support to deal with an incapacitating illness. Crucially, they are also gate keepers and advisers for relevant people and organizations who might provide support. Chances of support therefore often depend on encountering GPs, who consider their symptoms as legitimate, have knowledge of CFS/ME, actively support the client to diagnosis and beyond [54]. Our study also confirmed the importance of patients' belief in the medical encounter for building successful therapeutic clinical alliances, identified in studies such as Deale and Wesseley's [55], of patient perceptions of medical care in a specialist fatigue clinic,. Our findings confirmed the perceived consequences of such alliance-building for accessing further medical treatment and appropriate social and financial support.

Improving the by contrast, often-challenging, client-professional relationships was given priority in most participants' suggestions for policies and strategies, again, reflected in other studies [3,34,43,44]. In 2007, the UK Department of Health launched National Institute for Health and Clinical Excellence (NICE) guidelines for CFS/ME diagnosis and management [28]. While the comprehensiveness of these guidelines is still debated, aspects strongly supported by CFS/ME organisations and findings of this study include the importance of multidisciplinary and partnership working between healthcare professionals and patients, their families and carers. Setting up multidisciplinary teams across England is still in progress, with the role of primary care commissioners to ensure equitable provision of such services across England, remaining crucial.

In contrast to the NICE guideline recommendation that people with CFS/ME should be able to make informed decisions about their care and treatment [28], participants in this study indicated that their control over choices about health care remains in the hands of doctors many of whom appeared to lack the knowledge or ability to enable the personalised support needed. Fundamental care in diagnosis and treatment and diverse types of therapy and complementary care were valued as important (especially by members of ethnic minority groups) but not usually or sufficiently provided by the National Health Service, sometimes being accessible only to those in a position to pay but distancing them further from their GPs [27] Such differences contribute markedly to social inequalities in health for those in disadvantaged socio-economic conditions, who are less likely to receive the care they need. Advice on managing symptoms should be given from the outset, and not withheld until a diagnosis has been made. Healthcare professionals should proactively advise on flexible adaptations to work or study, referring to specialist CFS/ME care based on need. The benefits system as a whole needs procedures which can recognise the social support needs of those with cognitive impairments and communications cultural barriers for socially-disadvantaged groups including some ethnic minority groups.

The UK Department for Work and Pensions policy launched in 2008 [56], instituted a new benefit called Employment and Support Allowance (ESA) which aims to support disabled and ill people to return to work, by assessing what people can do rather than what they cannot. This encourages people lightly and moderately affected with CFS/ME to increase social participation by working. However, this study's findings indicate that the ESA's potential to improve equity in social care can only be fully realised if expert health professionals effectively assess their clients' potential, allowing for disease severity and fluctuation [57].

Policies and strategies should, therefore, focus on acknowledging the complexity of CFS/ME and the high intensity needs of people and families with this condition [58,59], enhancing professionals' expertise on CFS/ME, increasing coverage, personalisation and complexity of their care, ME, drawing on the expert opinions of service users, amplifying inclusion criteria, simplifying pathways and protocols to access care and benefits, and acknowledging human rights and disability rights. The study findings indicate that those policies and strategies suggested by people with CFS/ME sit well with evidence-based guidelines for overall management of their condition (19) and of chronic illnesses in general [60], as well as with wider policy recommendations for overcoming social inequalities in health [61] and for social inclusion of people with disabilities [62].

### Strengths and limitations

A strength of this study, is the diversity in professional and academic backgrounds of its authors, recognized to increase data analysis trustworthiness [36]. The relatively large purposive sample of 35 people, with greater diversity of social backgrounds and illness severity than many other studies of experiences of this illness, is also likely to have covered wider variations in experiences of the health and social services. The topic question guidelines for the data collection facilitated the emergence of story lines and of diverse needs. The data-led analysis, and the multiple trustworthiness mechanisms (including researcher and methodological and theoretical triangulation, respondent validation and member checking), increase the likelihood of ensuring that the findings represent participants' views [28,29]. Original contributions of this study include its overview of felt needs for support from health and social services and other social support, the identification of major sources of inequities in care and the priority policies and strategies suggested by people with CFS/ME.

A study limitation is its reliance on individuals' retrospective reports of experiences, and their fatigue, may, perhaps, have affected participants' ability to comprehensively communicate accounts of their needs. Another limitation is the lack of opportunity to test suggested strategies or solutions to improve such support and to decrease the financial plights encountered. However, an in-depth examination of best practice [37], and a detailed description of the pathways for health and social support (article in preparation), are among studies recently conducted by the CFS/ME Observatory to advance such knowledge. Further studies should explore how far these findings have resonance for a representative sample of people with CFS/ME and to compare ethnic groups' consultation and help-seeking behaviours and their impact on access to appropriate care for CFS/ME.

## Conclusions

People with CFS/ME in this study highlighted the complexity of their illness, its impact on well being and the intensity of their consequent needs to regain control over their lives. For health services to respond more equitably to these needs, they should provide earlier diagnosis, more holistic health-and social care management, and more coordinated, pro-active comprehensive support across services and support networks. These are vital to maintain social inclusion, secure practical support to manage life and to decrease the impact on carers and finances. Well-being was seen to depend on needs-based, personalised, provision which could acknowledge the expertise of people living with CFS/ME. In contrast, the experiences reported here consistently highlighted damaging cumulative health and social consequences of living with a life-disrupting long term condition that has been largely dismissed and stigmatised by care professionals, services and systems. For many, such consequences were amplified by socio-economic disadvantage, cultural and ethnic factors and lost work and civil status. The barriers to accessing health, social and financial support may be overwhelming for people who must often fight in isolation for their health and subsistence.

Study participants' experiences identified health and social policies and strategies needed to overcome these barriers to equity, by increasing wider understanding of high-intensity CFS/ME needs and their management, acknowledging the expertise of those living with the illness and developing a more inclusive care system. Enabling such policies was seen to require better-informed, more pro-active attitudes on the part of practitioners, policy makers and general public towards CFS/ME, advocacy, social justice and equitable care through investment in research and public and professional education.

A more equitable care system therefore requires more coordinated actions between people living with CFS/ME, service providers, policy makers and public, private and voluntary organizations for people with CFS/ME and for human rights. Further research to explore the resonance with a larger group of people with CFS/ME, of these indicative findings on the importance of social support for equity, is now needed.

In summary, the study has highlighted the damaging bio-psychosocial effects of living with CFS/ME within a system which has engendered health and social care provision which is socially-unsupportive of their specific needs. Its findings suggest for health practice and future studies that, as Marmot and Friel urge [1], as well as acting to understand and manage the illness, creative responses to inequity should draw on joint ventures with communities of people with CFS/ME, their carers and families, health professionals and social policy makers. Where individuals are affected by long-term, fluctuating conditions with complex needs, as exemplified in CFS/ME, health professionals should be especially prepared to act as advocates for patients in negotiations with employers, educational and social welfare organisations to respond flexibly if more equity in health and social inclusion is to be enabled for these groups.

## Competing interests

The authors declare that they have no competing interests.

## Authors' contributions

JL, LN, DP conceived the study; JL, LN, MD, MM and FP designed the methodology and JL, MD, LN, DP and FP worked to gain ethical clearance from London MREC; MD organised the process of recruitment,. JL, MD, CH and FP recruited the subjects. MM and JL conducted the focus group and JL conducted the one-to-one interviews. JL coordinated the data analysis and initial writing up of the article; JL, AK and SK analysed the data; MM, FP, AK and SK organised the validation exercises and dissemination events; JL, AK, MD, SK and FP wrote the first draft of the article. FP coordinated the final writing up of the article.. All authors contributed to the manuscript and approved the final version.

## Authors' Information

School of Allied Health Professions, University of East Anglia, Norwich NR4 7TJ, UK

FP, Senior Lecturer in Health and Society and Joint Principal Investigator for the CFS/ME Observatory; AK, Lecturer in Occupational Therapy, CSH, Lecturer in Occupational Therapy; SK, Lecturer in Physiotherapy; MdeLD, Reader in Health Equity and Social Inclusion and Joint Principal Investigator for the CFS/ME Observatory; JL, Senior Research Associate in Social Research; MM, MSc Pre-registration OT Programme Director, LO'D, Senior Lecturer in Physiotherapy.

*Postgraduate Medical Institute, University of Hull, Daisy Building, Castle Hill Hospital, Castle Road, Cottingham, East Yorkshire, HU16 5JQ*.

PC, Emeritus Professor of Primary Care Medicine

*London School of Hygiene and Tropical Medicine, Keppel Street, London WC1E 7HT, UK*.

LN, Clinical Lecturer; EL, Research Fellow

*Faculty of Society and Health, Buckinghamshire New University, Uxbridge Campus, 106, Oxford Road, Uxbridge, Middlesex, UB8 1NA*.

DP, Visiting Professor of Epidemiology
